# Epileptogenic but MRI-normal perituberal tissue in Tuberous Sclerosis Complex contains tuber-specific abnormalities

**DOI:** 10.1186/s40478-015-0191-5

**Published:** 2015-04-02

**Authors:** Alexander A Sosunov, Robert A McGovern, Charles B Mikell, Xiaoping Wu, David G Coughlin, Peter B Crino, Howard L Weiner, Saadi Ghatan, James E Goldman, Guy M McKhann

**Affiliations:** Departments of Neurological Surgery, Columbia University Medical Center, NI-42, 710 West 168th street, New York, NY 10032 USA; Departments of Neurology, Temple University School of Medicine, Philadelphia, PA 19140 USA; Departments of Neurological Surgery, New York University Medical Center, New York, NY 10016 USA; Departments of Neurosurgery, Mount Sinai Hospital, New York, NY 10029 USA; Departments of Pathology & Cell Biology, Columbia University, New York, NY 10032 USA

**Keywords:** Tuberous sclerosis complex (TSC), Perituberal area, Cortical tuber, Microtuber, Astrogliosis

## Abstract

**Introduction:**

Recent evidence has implicated perituberal, MRI-normal brain tissue as a possible source of seizures in tuberous sclerosis complex (TSC). Data on aberrant structural features in this area that may predispose to the initiation or progression of seizures are very limited. We used immunohistochemistry and confocal microscopy to compare epileptogenic, perituberal, MRI-normal tissue with cortical tubers.

**Results:**

In every sample of epileptogenic, perituberal tissue, we found many abnormal cell types, including giant cells and cytomegalic neurons. The majority of giant cells were surrounded by morphologically abnormal astrocytes with long processes typical of interlaminar astrocytes. Perituberal giant cells and astrocytes together formed characteristic “microtubers”. A parallel analysis of tubers showed that many contained astrocytes with features of both protoplasmic and gliotic cells.

**Conclusions:**

Microtubers represent a novel pathognomonic finding in TSC and may represent an elementary unit of cortical tubers. Microtubers and cytomegalic neurons in perituberal parenchyma may serve as the source of seizures in TSC and provide potential targets for therapeutic and surgical interventions in TSC.

## Introduction

Cortical tubers are a hallmark of the brain pathology and a main source of epileptic activity in patients with tuberous sclerosis complex (TSC) [1]. TSC is an autosomal dominant disorder caused by mutations in *TSC1* or *TSC2*. Loss of function of these genes leads to activation of the mTOR cascade, which is critical for cellular growth and development of numerous organ systems [2-4].

Tubers are a type of cerebral cortical malformation that are characterized by a prominent irregularity in neocortical cytoarchitecture. Tubers are composed of abnormal cells including giant cells, dysplastic/cytomegalic neurons, and aberrant tuber astrocytes. Although tubers are generally thought to be a source of ictal activity in TSC epilepsy, seizure initiation from perituberal tissue has been documented in several reports suggesting other mechanisms for epileptogenesis than the effects of tubers on network excitability [5-9].

Perituberal “MRI-normal” tissue is often resected during TSC epilepsy surgery as part of the epileptogenic zone, and is usually considered to be a structurally “normal” area of brain adjoining a nearby epileptogenic cortical tuber. For example, Wang et al. [10] reported an epileptogenic focus in a TSC patient who displayed no cortical tubers by MRI. However, analysis of gliosis in TSC revealed perituberal collections of giant cells and gliotic astrocytes that we designated “microtubers” [11]. A recent autopsy study also detected considerable TSC pathology outside of cortical tubers, including lamination abnormalities, heterotopias, and giant cells [12]. These authors also designated pathological clusters of giant cells and dystrophic neurons outside of tubers as “microtubers.”

In this study, we have examined MRI-normal, perituberal tissues in patients who had surgical resections for epilepsy. The resected tissue included perituberal tissue as part of the epileptogenic zone in many of these patients. In every sample of epileptogenic, perituberal tissue, we found abnormal cellular pathology, including cytomegalic neurons, giant cells, and gliotic astrocytes. Most of the perituberal giant cells or groups of giant cells in the cortex were surrounded by gliotic astrocytes, together forming “microtubers”. We fully describe the features of perituberal “microtubers” and focus on the importance of pathological gliotic astrocytes together with giant cells as the fundamental elements of these potentially epileptogenic structures.

## Materials and Methods

### Tissue

Specimens including cortical tubers (n = 16) and MRI normal perituberal tissue (n = 10) were obtained from TSC patients with medically refractory epilepsy who were undergoing epilepsy surgical resection. In one case, hippocampus with MRI normal architecture was resected as the epileptogenic area together with adjoining cortical tubers. The mean age of the 16 patients was 9.8 ± 1.5 years (range: 2.5 – 23 years), with 9 females and 7 males. The epileptogenicity of the resected tubers and perituberal tissue was established during invasive subdural intracranial monitoring of patients, as previously described [9]. Perituberal tissue did not show any abnormalities on detailed preoperative MRI examination using imaging protocols designed to maximize preoperative detection of malformations of cortical development. The location of the tuberal tissue and MRI normal perituberal tissue was confirmed intraoperatively by gross visual inspection, as well as by frameless stereotactic intraoperative MRI based surgical navigation. All patient protocols were approved by the Institutional Review Boards of Columbia University Medical Center and New York University Medical Center.

### Histology and immunohistochemistry

Specimens were fixed in 4% paraformaldehyde in PBS for 14–18 h (4°C). 40 μm sections were prepared with a vibratome (Leica VT1000S) and stored in cryoprotectant solution at -20°C. Standard procedure for Nissl staining with cresyl violet was used for routine analysis of tissue.

### Antibodies

Primary antibodies used in the study are shown in Table 1.

**Table 1:** **Primary antibodies used for immunohistochemistry**

Glial fibrillary acidic protein (GFAP)	Monoclonal mouse	Sigma-Aldrich, St. Louis, MO	1:1000
Glial fibrillary acidic protein (GFAP)	Polyclonal rabbit	DAKO, Carpinteria, CA	1:1000
Glial fibrillary acidic protein (GFAP)	Polyclonal chicken	Covance, Berkeley, CA	1:500
Vimentin	Monoclonal mouse	DAKO	1:500
Nestin	Monoclonal mouse	Covance	1:500
Glutamine synthetase	Monoclonal mouse	MIllipore, San Diego, CA	1:1000
Glutamine synthetase	Polyclonal rabbit	Santa Cruz Biotechnology, Santa Cruz, CA	1:200
EAAT1 (GLAST)	Monoclonal mouse	Novocastra Lab, Newcastle upon Tyne, UK	1:100
EAAT2 (GLT-1)	Polyclonal guinea-pig	Millipore	1:1000
EAAT2 (GLT-1)	Polyclonal rabbit	Cell Signaling Technology, Inc, Boston, MA	1:100
S100	Polyclonal rabbit	DAKO	1:400
CD44	Monoclonal mouse	DAKO	1:80
CD44	Monoclonal rat	Millipore	1:200
alphaB-Crystallin	Polyclonal rabbit	Stressgen, Canada	1:300
SPARC/Osteonectin	Polyclonal goat	R&D Systems, Inc., Minnezpolis, MN	1:200
SPARC/Osteonectin	Polyclonal rabbit	Cell Signaling	1:200
MAP2	Monoclonal mouse	Sigma-Aldrich	1:250
Pan-neuronal neurofilament marker SMI 311	Monoclonal mouse	Covance	1:1000
NeuN	Monoclonal mouse	Millipore	1:100
CD68	Monoclonal mouse	DAKO	1:300
LN-3	Monoclonal mouse	ICN Biomedicals, Inc., Aurora, OH	1:50
Iba-1	Polyclonal rabbit	Biocare Madical, LLC, Concord, CA	1:500
Phospho-S6 ribosomal protein (Ser 235/236)*	Monoclonal rabbit	Cell Signaling	1:200
Phospho-S6 ribosomal protein (Ser 240/244)*	Monoclonal rabbit	Cell Signaling	1:200
Phospho-p44/42 MAPK (Erk1/2) (Thr202/Tyr204)	Monoclonal rabbit	Cell Signaling	1:100
Tumor necrosis factor alpha (TNF alpha)	Polyclonal goat	Santa Cruz	
Neurotropin receptor p75NTR	Polyclonal rabbit	Promega Corp.,Madison, WI	1:500

*- these two antibodies gave similar results.

Secondary antibodies include: conjugated to fluorophores: anti-mouse Alexa Fluor 488, 594, and 633; anti-chicken Alexa Fluor 594, 633; anti-rabbit Alexa Fluor 594; anti-goat Alexa Fluor 594, 633; anti-guinea pig Alexa Fluor 594, 633, all from goat or donkey (1:300, Molecular Probes, Eugene, OR).

Antigen retrieval procedure was performed with Antigen Unmasking Solution (Vector Laboratories, Inc., Burlingame, CA) by boiling for 40 sec in a microwave.

For light microscopy sections were pretreated in 3% H_2_O_2_ in methanol (30 min, at room temperature [RT]) and after blocking in 10% donkey serum (30 min, RT) incubated overnight (4°C) with primary antibodies. After washing sections were incubated with biotinylated secondary antibodies (1:1000, 1 hr, RT) and then, after washing, with avidin–biotin-peroxidase complex (ABC, Vector Lab.) (1 hr, RT). Peroxidase activity was visualized with 0.03% DAB (Sigma) with 0.005% H_2_O_2_ in 0.05 M Tris buffer. Sections were transferred to glass slides, dehydrated in a graded ethanol series and mounted in DPX (Sigma).

For double- and triple-immunofluorescence, after blocking with 10% normal goat (or donkey) serum (30 min, RT), free-floating sections were incubated in a mixture of primary antibodies raised in different species for overnight (4°C). For visualization, Alexa Fluor-conjugated secondary antibodies were used for 1 h at RT. Fluorescent Nissl reagent (NeuroTrace 640/660 deep-red, 1:150, Molecular Probes) was applied simultaneously with secondary antibodies in cases of double-immunostaining for visualization of general histological structure.

Blocking serum, primary, secondary antibodies, and fluorescent Nissl reagent were applied in 0.2 % Triton X-100 in PBS. Sections for fluorescent microscopy were mounted on slides in Vectashield (Vector Lab). To control the specificity of immunostaining, primary antibodies were omitted and substituted with appropriate normal serum. Slides were viewed using a confocal microscope (Bio-Rad Radiance 2000, Nikon E800).

### Quantitative immunohistochemical analysis

We determined the size of microtubers using the diameter of the area occupied by astrocytes with high levels of GFAP or vimentin in the images (merged from the stacks of adjacent 6 images [1024 × 1024 pixel resolution, observed area 606 × 606 μm] captured at a distance of 1 μm from each other) obtained with a confocal microscope. In cases of oval-shaped microtubers, the longest diameter was measured. The border of microtubers was considered as the point at which the immunostaining of GFAP/vimentin decreased significantly. The long processes of astrocytes were excluded in the determination of borders. Furthermore, since most tubers contained astrocytes that did not stain with anti-GS antibodies, a double immunostaining for GS and either GFAP or vimentin showed the border clearly.

The numbers of (1) giant cells (detected with GS and p-S6 immunostaining), (2) p-S6+, p44/42+, and SPARC+ astrocytes in microtubers (detected in double immunostained sections for p-S6/p44/42/SPARC and GFAP), (3) CD68- and IBA1-immunolabeled microglial cells (in microtubers in double immunostaining with GFAP, and in tuber border area in double immunostaining with GS) were quantified in the images merged from a stacks of adjacent 6 images (1024 × 1024 pixel resolution, observed area 606 × 606 μm) captured at a distance of 0.5 μm from each other. Only cells with clearly outlined nuclei were counted.

The size of a giant cells was quantified based on OD of the area occupied by the cell body immunoreactive for p-S6 and GS. In perituberal areas, the data on giant cells in microtubers and giant cells located in normal parenchyma were pooled together because we did not find significant differences between these types of cells. Images were obtained from stacks of 6 images (1024 × 1024 pixel resolution, observed area 148 × 148 μm, step 0.5 μm). Only those cells that were unequivocally indentified as giant cells (based on a large size, irregular shape, and immunostaining) were taken into consideration.

To determine the level of GS, CD68 and Iba1 immunoreactivity, optical density (OD) was measured in the area of interest (in microtubers, occupied with reactive astrocytes with high levels of GFAP) in the images prepared as above (area of observation was 295 × 295 μm) that were transferred to Adobe Photoshop, grayscaled and used for analysis of OD with Scion Image Beta 4.02 (public domain). Level of EAAT2 immunoreactivity was measured based on OD obtained from merged stacks of adjacent 6 images (1024 × 1024 pixel resolution, observed area 148 x 148 μm) captured at a distance of 0.25 μm from each other). EAAT2 and GFAP immunoreactivities correspond to different cellular compartments (cytoplasm and plasma membrane, respectively) and the surface occupied by GFAP and EAAT2 inversely correlate (more cytoplasm in the area used for analysis of OD, less plasma membranes). To determine the OD of EAAT2 we used an equation: OD_EAAT2_ = OD_EAAT2_/(S_AOI_ − OD_GFAP_ − OD_Nissl_), where S_AOI_ stands for the area of the image where OD was determined, OD_GFAP_ and OD_Nissl_ stand for area occupied with GFAP OD and nuclei, respectively. Thus we have calculated arbitrary units, that indicate the EAAT2 OD for arbitrary units of plasma membrane.

More than 6 images were used from each case from every area of interest (perituberal area, microtubers, tubers, tuber borders) for quantification of cell number and OD.

### Statistical analysis

Data are expressed as mean ± SEM. Differences in the incidence were analyzed by Chi-square test. Continuous parameters were analyzed with Student’s *t*-test or with one- way ANOVA where appropriate. P < 0.05 was considered significant.

## Results

### Defining the borders of cortical tubers

**Figure 1:**
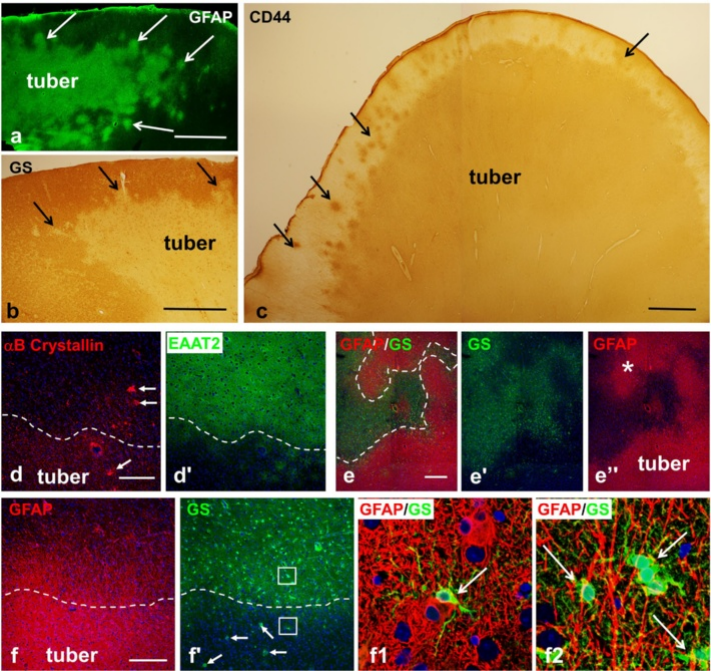
**Astrogliosis delineates the border of cortical tubers. (a-c)** High levels of GFAP **(a)** and CD44 **(c)** and low levels of glutamine synthetase (GS) **(b)** outline tubers from surrounding normal tissue. Note undulated surface of the tubers and appearance of small gliotic foci (microtubers) nearby (arrows, marked only some). **(d)** Gliotic tuber astrocytes reveal a profound decrease in EAAT2 and high levels of αB-Crystallin. Note: giant αB-Crystallin immunopositive cells (arrows) are present in tuber and in perituberal area. **(e)** A difference in the levels of GS and GFAP outline the main body (tuber) and gliotic protuberances (*) which make tuber border highly irregular in shape. **(f)** Protoplasmic (high levels of GS) and gliotic (high levels of GFAP) astrocytes are neatly segregated at a tuber border (dotted line). Note that giant cells (arrows) show high level of GS expression. **(f1)** (lower boxed area outlined in **f)** Only few protoplasmic astrocytes (arrow indicates one protoplasmic astrocyte) are located within the gliotic tuber tissue near the border. **(f2)** (upper boxed area outlined in **f)** Long processes of tuber astrocytes penetrate into the perituberal area populated with protoplasmic astrocytes (arrows). **(d-f)** Confocal microcopy, double immunostaining, counterstaining with Nissl. **d**’, **e**’, **e**”, and **f**’ represent split **d**, **e**, and **f** images, respectively. In **d** and **d**’ only split images obtained from double-immunostained sections are shown. Tuber border is depicted with dotted line. *scale bars*: 950 μm in **a**-**c**; 150 μm in **d**-**f**.

To discriminate between perituberal cortex and tubers, it is first critical to demarcate the cortical tubers themselves in histological sections. Based on immunohistochemical analysis, we have found that astrogliosis is the best criterion for morphological discrimination between tubers and the surrounding neocortical parenchyma (Figure 1). In addition to providing a clear histological delineation of tubers, this proposal is in line with MRI data where both T1- and T2-weighted images correlate with the level of tissue sclerosis/gliosis [13]. Several markers of astrocytes were used to determine tuber astrogliosis and accordingly tuber borders. These included markers typical for gray matter protoplasmic astrocytes such as glutamine synthetase (GS) and the astrocyte specific glutamate transporters (EAAT1 and EAAT2), all of which showed profoundly lower levels in tubers (Figure 1). In addition, markers that are seen in reactive/gliotic astrocytes such as glial fibrillary acidic protein (GFAP), vimentin, CD44, αB-Crystallin, and S100 revealed striking increases in tuber tissue (Figure 1, only some shown). Tuber borders were thus clearly outlined, segregating perituberal protoplasmic astrocytes from tuber gliosis (Figure 1). We observed that the borders of tubers were not always smooth, displaying uneven profiles with many peripheral protrusions (Figure 1a,b,c,e). Long processes of tuber astrocytes penetrated into the perituberal parenchyma and intermingled with neighboring protoplasmic astrocytes (Figure 1f). Furthermore, small foci of astrogliosis (microtubers, see below) were regularly found near the tuber borders (Figure 1a,b,c).

### Tuber-specific cellular abnormalities in perituberal MRI-normal tissue

Most of the observations presented in this section were obtained from MRI-normal, epileptogenic perituberal tissue (n = 10) that was dissected during surgery separately from tubers. Macroscopic tubers were never found in these samples of tissue.

#### Microtubers are common pathological feature of perituberal tissue

**Figure 2:**
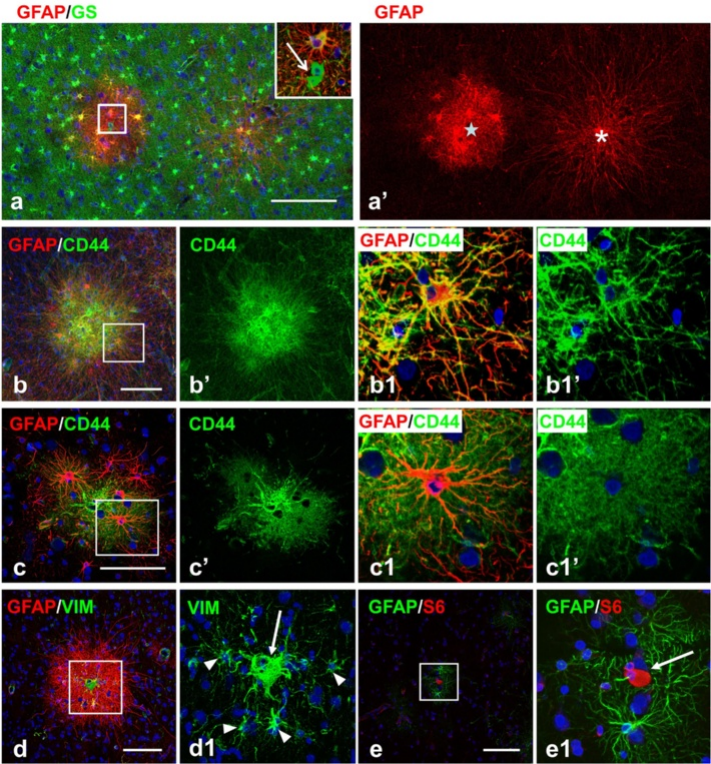
**Microtubers in perituberal tissue. (a) **Microtubers differ in the shape of astrocytes: type I (asterisk) has astrocytes with long processes, whereas type II (star) has astrocytes with regular lengths of processes. Inset, enlarged boxed area outlined in (a), GS+ giant cell (arrow) in type II microtuber. **(b, c)** Immunostaining for plasma membrane glycoprotein CD44 emphasizes the difference in the shapes of astrocytes in type I **(b)** and type II **(c)** microtubers. Note that fibrous-like astrocytes in type I microtubers do not have small leaf-like processes and main branches are clearly outlined **(b1)**, in contrast CD44+ astrocytes in II type microtubers, display an abundance of miniature leaf-like processes located on main branches, which produces the characteristic bushy-like view of the cell **(c1)**. **(d)** Type I microtuber with vimentin (VIM) immunoreactive giant cell (arrow). Note that astrocytes (arrowheads) with long process express VIM, a typical feature of reactive astrocytes. **(e)** Microtuber composed of a p-S6+ giant cell (arrow) surrounded only by a few astrocytes with high levels of GFAP. Confocal microscopy, double immunostaining, counterstaining with Nissl. **a**’, **b**’,**b1**’, **c**’, and **c1**’represent split images of **a**, **b**, **b1**, **c**, and **c1**, respectively. **b1**, **c1**, **d1**, and **e1** –enlarged boxed area outlined in **b**, **c**, **d**, and **e** respectively. *scale bars*: 150 μm in **a**; 55 μm in **b**,**c,** and **d**; 80 μm in **e**.

In contrast to the highly gliotic tubers, the perituberal gray matter was populated mainly with protoplasmic astrocytes displaying high levels of GS and low levels of GFAP (Figure 2a,b). To detect abnormal cells typical for tubers within perituberal tissue, we used several markers specific for neurons and glial cells and known to be increased in giant cells and cytomegalic neurons [14,15], as well as p-S6, to look for mTOR activation [16]*.* In every section from every specimen of perituberal tissue, we found aberrant cells characteristic of tubers: giant cells, gliotic astrocytes, and cytomegalic neurons.

In the perituberal gray matter, giant cells were usually surrounded by fibrous-like astrocytes with long processes displaying high levels of GFAP and CD44. These small astrogliotic islands were clearly outlined from the neighboring normal parenchyma containing protoplasmic astrocytes (Figure 1a,b,c; 2). We previously designated these microscopic islands as “microtubers” [11], in contrast to the canonical cortical “macrotubers” detected by MRI. They were relatively homogeneous in size, with an average diameter of 284.7 ± 17.3 μm (n = 70; range: max 461.6 μm, min 158.6 μm). If we consider the shape of microtubers as roughly spherical, we estimate that one microtuber is composed, on average, of ~ 27 astrocytes and includes ~ 20 neurons.

Two types of microtubers were distinguished based on the shapes and immune profile of astrocytes. The majority (∼80%) of microtubers (which we designated as type I) contained many astrocytes with long processes that radiated for several hundred micrometers into the adjacent gray matter, which was occupied by protoplasmic astrocytes (Figure 2a,b,d). The processes of these cells were devoid of the miniature lamellipodial-like processes that are a characteristic feature of protoplasmic astrocytes. Such structural feature was especially obvious when cells were immunostained for the plasma membrane glycoprotein CD44 (Figure 2b1). A minority (~20%) of microtubers (designated type II) were largely composed of astrocytes with processes of normal length endowed with many miniature lamellipodial leaf-like extensions that produced the typical bushy-like appearance of protoplasmic astrocytes (Figure 2a,c). However, in contrast to typical protoplasmic astrocytes, these cells were CD44+ (Figure 2c). It is worth noting that some type II microtubers contained only a few (2–4 in a plane of inspection) reactive-like astrocytes neighboring a giant cell (Figure 2e).

**Figure 3:**
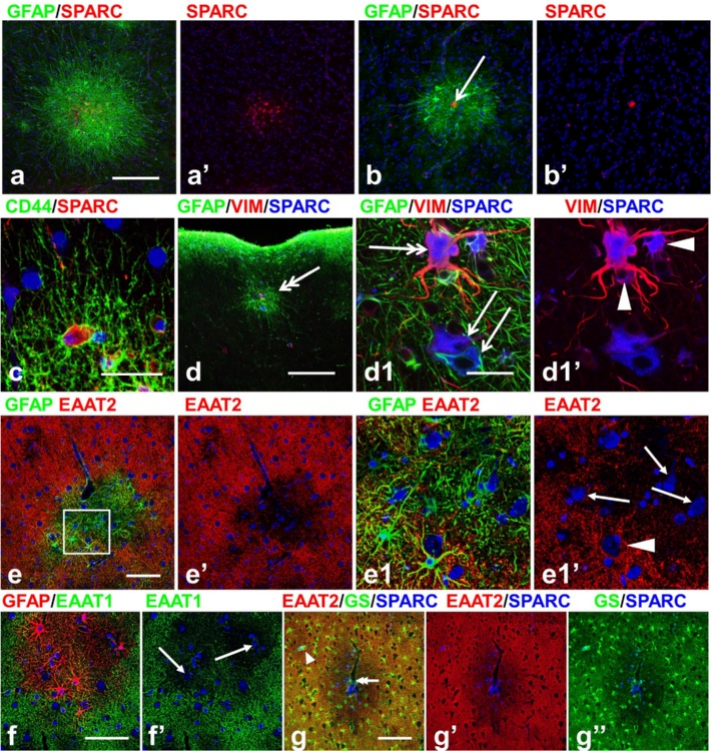
**Astrocyte properties in microtubers. (a)** Type I microtuber contains many SPARC+/CD44+ astrocytes with long processes. **(b)** Astrocytes in type II microtuber are SPARC-negative. Note SPARC+ giant cell (arrow) in **b**). **(c)** SPARC+/CD44+ long process astrocyte does not have miniature lamellipodial-like processes. **(d,d1)** Type I microtuber in upper cortical layers with vimentin+/SPARC+ (double headed arrow) and SPARC+ (arrows) giant cells. Note that astrocytes (arrowheads) near giant cells are SPARC+ and vimentin+. **(d1)** enlarged central part of microtuber in **d**). **(e-g)** Glutamate transporters (both, EAAT1 and EAAT2) and GS are decreased in microtuber astrocytes. Note that the central parts of microtubers with many neurons (arrows) are depleted of EAAT2 **(e,** **e1)**, EAAT1 **(f)**, and GS **(g)**. Giant SPARC+ cell located in the central part of the microtuber near blood vessel is marked with double-headed arrow **(g)**. Isolated SPARC+/GS+ giant cell in the neighboring parenchyma is marked with arrowhead **(g)**. Confocal microscopy, double immunostaining **(a-f)**, counterstaining with Nissl, triple immunostaining **(g)**. **a1**–enlarged boxed area outlined in **a**. **a**’, **b**’**, e**’, **e1**’, **f**’, **g**’, and**g**”represent split images from **a**, **b**, **e**, **e1**, **f**, and **g**, respectively. *scale bars*: 100 μm in **a**,**b**; 45 μm in **c**,**d**; 115 μm in **e**,**f**; 170 μm in **g**.

**Figure 4:**
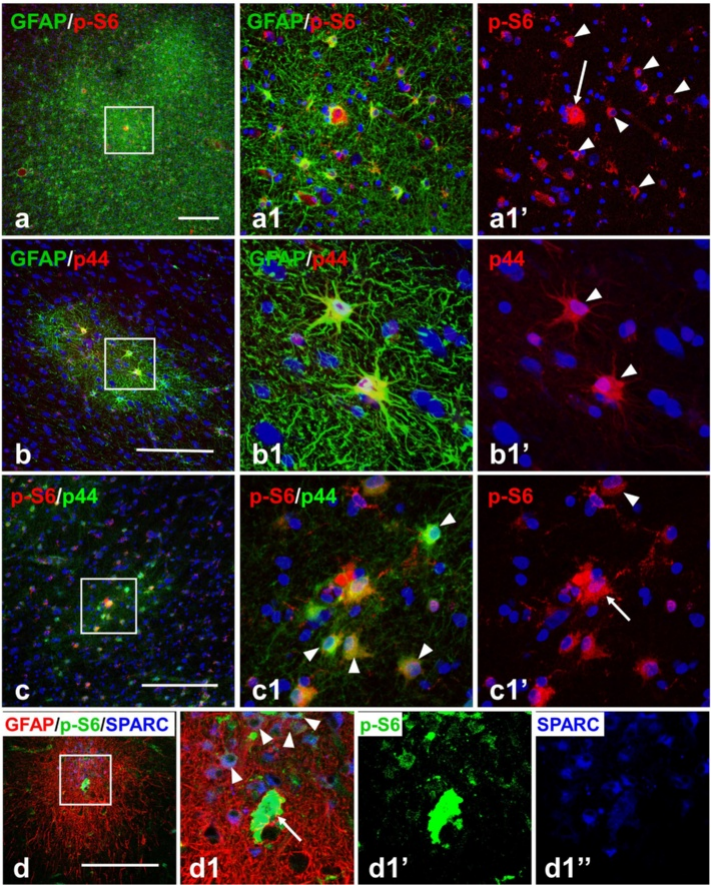
**Astrocytes in microtubers reveal activation (phosphorylation) of ribosomal protein S6 (S6) and p44/42 MAPK (p44). (a)** p-S6+ astrocytes (arrowheads, marked only some) in microtubers in cortical layer V. A giant p-S6+ cell is marked with an arrow. Note that several microtubers are located near each other. **(b**,**c) **p44+ astrocytes (arrowheads) in microtubers detected with polyclonal (**b**, red) and monoclonal (**c**, green) primary antibodies. Giant cell p–S6+ and p44+ is marked with an arrow. **(d)** Many SPARC+ fibrous astrocytes express p-S6 (arrowheads). Giant p-S6+ /SPARC+ cell is marked with an arrow. Confocal microscopy, double immunostaining, counterstaining with Nissl **(a-c)**, triple immunostaining **(d)**. **a1**, **b1**, **c1**, and **d1**–enlarged boxed area in **a**, **b**, **c**, and **d**, respectively. **a1**’, **b1**’, **c1**’,**d1**’ and **d1**”represent split **a1**, **b1**, **c1**, and **d1** images, respectively. *scale bars*: 150 μm.

We suggest that the astrocytes with long, non-branched processes in type I microtubers are similar in many ways to the CD44+ long-process/interlaminar astrocytes in gray matter and/or to fibrous astrocytes in white matter, whereas astrocytes with processes of protoplasmic astrocytes size and shape, but CD44+, in type II microtubers are reactive protoplasmic astrocytes. To test this hypothesis we used immunostaining for SPARC/osteonectin, a glycoprotein we have found to be a characteristic marker of CD44+ interlaminar and fibrous astrocytes in human brain [17]. Indeed, type I microtubers contained many SPARC+ astrocytes whereas only a few SPARC+ cells were observed in type II microtubers (11.8 ± 0.824 per microtuber in type I vs 0.824 ± 0.3 in type II, p < 0.001) (Figure 3a,b). In addition, all SPARC+ astrocytes were CD44+ and had clearly outlined, long main branches without lamellipodial-like processes (Figure 3c). It should be noted that many giant cells also showed immunolabelling for SPARC (Figures 3b,d; 4c,d).

Astrocytes in both types of microtubers were characterized by diminished immunoreactivity for glutamate transporters (EAAT2 and EAAT1) (only EAAT2 was quantified, OD, arbitrary units, 0.1 ± 0.03 in microtubers vs 0.6 ± 0.3 in surrounding normal parenchyma, p < 0.001) and for GS (OD, arbitrary units, 1199.7 ± 296.6 in microtubersvs 9874.4 ± 1321.8 in surrounding normal parenchyma, p < 0.001) (Figure 3e-g). This decrease in GS and glutamate transporters was particularly prominent in the central part of microtubers, where many neurons were surrounded by astrocytes lacking these proteins (Figure 3e-g).

Many astrocytes in microtubers showed p-S6 immunoreactivity (Figure 4a,c,d). Especially prominent p-S6 expression was observed in microtubers located in deep cortical layers (255 of 349 astrocytes in V-VI layers vs. 46 of 123 astrocytes in upper I-IV layers, p < 0.001).

To gain additional insight into the characteristics of astrocytes in microtubers, we tested activation (phosphorylation) of p44/42 MAPK (p44), which was previously shown to be activated in tubers [18]. We found that many astrocytes in microtubers displayed a high level of p44 (Figure 4b,c). Microtubers located in deep cortical layers and in white matter contained more p44+ astrocytes than those in upper gray matter layers (56 of 121 cells in upper layers vs. 156/184 in deep layers, p < 0.001). Only a few (6/61) giant cells revealed immunoreactivity for p44 (Figure 4c). Many p44+ astrocytes were also immunopositive for p-S6 (in deep cortical microtubers ~ 80%, 214 of 305 p44+ astrocytes) (Figure 4c). Many SPARC+ astrocytes were also p-S6+ (Figure 4d).

**Figure 5:**
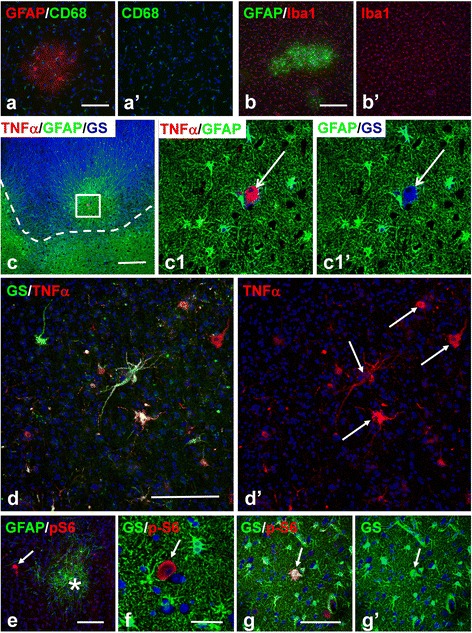
**Microglia and giants cells in micro- and macrotubers.** **(a,b)** Microglia are not activated in microtubers, levels of CD68 **(a)** and Iba1**(b)** immunoreactivity are not increased within microtubers. **(c,d)** Immunolabelling for tumor necrosis factor alpha (TNFα) in microtubers **(c)** and in tubers **(d)**. **(c)** I type microtuber located near the tuber (marked with a dashed line) with a giant cell (arrow in **c1** and **c1**’) both GS+ (blue) and TNFα + (red). **(d)** Many giant cells (arrows, marked only some) contain TNFα in the central part of the tuber. **(e-g)** Giant cells (arrows) in perituberal area located within non-reactive protoplasmic astrocytes. Note that in **e)** giant cell (arrow) is located near microtuber (asterisk). Confocal microscopy, double immunostaining **(a,b,d-g)** counterstaining with Nissl, triple immunostaining **(c)**. **a**’, **b**’,**d**’,**c1**’,**g**’ represent split **a**, **b**, **d**,**c1**,**g** images, respectively. **c1** - enlarged boxed area outlined in **c**. *scale bars*: 100 μm in **a**-**c**,**e**-**g**; 75 μm in **d.**

The appearance of reactive-like astrocytes in the immediate vicinity of giant cells in microtubers raised the possibility that giant cells might modulate their environment. We examined microtubers for microglial reaction as a marker of inflammatory brain parenchymal disturbances. In 45/47 microtubers tested with CD68 and LN-3 immunostaining, there was no prominent activation of microglia detected (shown only CD68, Figure 5a). Optical density of CD68 immunolabelling did not differ in microtubers from surrounding normal parenchyma (6150 ± 885 arbitrary units per 1 mm^2^ in microtubers vs 6030 ± 799 in normal parenchyma, P = 0.922). In only 2/47 microtubers, activated microglia were observed in large areas that included the microtubers but extended far beyond their boundaries (not shown). We also used immunostaining for Iba1, as a general marker for both resting and activated microglia, and did not find significant microglial changes in microtubers (Figure 5b). Numbers of Iba1+ microglial cells did not differ significantly from surrounding normal parenchyma (61.3 ± 7.66 cell per 1 mm^2^ in microtubers vs 55.5 ± 8.08 in normal parenchyma, P = 0.62).

We assayed also whether giant cells contain tumor necrosis factor alpha (TNFα), as shown previously [18]. TNFα is a cytokine that is responsible for induction of reactive changes in astrocytes in variable brain pathologies [19]. We found that many giant cells were TNFα+ (in microtubers 14 of 21 cells, in macrotubers 77/115, p = 0.722) (Figure 5c,d).

In perituberal tissue, in addition to giant cells in microtubers, we also found scattered giant cells surrounded by normal appearing protoplasmic astrocytes (Figure 5e,f,g). There were <1/10^th^ as many of these isolated giant cells in comparison to giant cells within microtubers (24 versus 235 cells), and they were found predominantly in upper (I – III) cortical layers (19 of 24).

**Figure 6:**
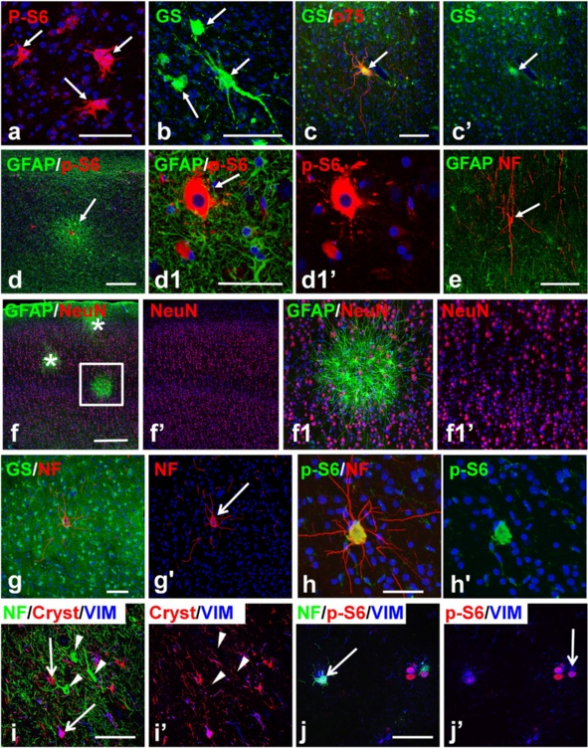
**Giant cells and cytomegalic neurons in tubers and microtubers.** **(a-c)** Giant cells (arrows) in tubers have large size, irregular shape, and long processes. Note in **c)** giant cell is immunopositive for p75. **(d,e)** Tuber-specific abnormalities in epileptogenic hippocampus in TSC. **(d)** Microtuber (arrow) in CA1 subfield. **d1** enlarged central part of the microtuber in **d**) with giant cell (arrow); **(e)** Cytomegalic neuron (arrow) in CA1 subfield. **(f)** Laminar cytoarchitecture of the cortex visualized with NeuN immunostaining is not changed in the microtubers (marked with asterisks and boxed area). **f1)** enlarged boxed area in **f)**. **(g)** Cytomegalic neuron (arrow) visualized by immunostaining for neurofilaments (NF) within normal appearing parenchyma populated with protoplasmic astrocytes. **(h)** Cytomegalic neuron immunopositive for p-S6. Note: level of neurofilament immunostaining in cytomegalic neurons exceeds that in surrounding normal neurons **(g,h)**. **(i)** Cytomegalic neurons (arrowheads) located in the upper cortical layers in the tuber don’t express markers (vimentin [VIM] and alpha B Crystallin [Cryst]) of giant cells (arrows). **(j)** Giant cells located in the tuber at a level of the white matter are immunopositive for neurofilaments (arrows). Confocal microscopy, double immunostaining **(a-h)**, counterstaining with Nissl, triple immunostaining **(i,j)**. **c**’, **d1**’, **f**’, **f1**’**, g**’, **h**’**, i**’, and **j1**’ represent split **c**, **d1**, **f**, **f1**, **g**, **h**, **i**, and **j1** images, respectively. *scale bars*: 100 μm in **a**-**e**; 150 μm in **f**-**h**; 80 μm in **i**,**j**.

Giant cells in the perituberal gray matter (both in microtubers and isolated) never reached as large a size as those within tubers (374.3 ± 22.2 μm^2^ (n = 20) vs 888.9 ± 40.88 μm^2^ (n = 20), respectively, p < 0.001) and never had as long and thick processes as giant cells in tubers (compare those in Figure 5e-g with those in Figure 6a-c). We also did not find giant cells immunopositive for p75 neurotrophin receptor (p75) in perituberal tissue although p75+ giant cells were often observed in tubers (106/178 in tubers vs. 0/34 in microtubers, p < 0.001) (Figure 6c). Such differences in giant cells might indicate different developmental routes or different growth and ‘maturation’ of giant cells.

An examination of a single TSC patient’s hippocampus that had a normal MRI appearance but was resected as part of the epileptogenic zone revealed microtubers and cytomegalic neurons in Ammon’s horn (Figure 6d,e), as well as in the hilus (not shown). This further supports the idea that microtubers represent important parts of epileptogenic zones apart from tubers.

#### Cytomegalic neurons in perituberal tissue

Analysis of specimens stained with the pan-neuronal marker NeuN did not show any peculiar features of the neuronal population (cellular density, cytoarchitecture, aberrant cellular types) in microtubers (Figure 6f).

Cytomegalic/dysplastic neurons similar to those characteristically found in tubers were also consistently found in perituberal gray matter. These cells were morphologically identified as large cells with long processes that were immunopositive for neuronal (neurofilaments [NF], microtubule-associated proteins [MAP2], and NeuN) markers and immunonegative for giant cell markers (GS, vimentin, nestin) (Figure 6g,h). Many giant cells in perituberal tissue and tubers were also positive for NF and/or MAP2 (Figure 6i,j, shown only NF). However, in contrast to cytomegalic neurons, the NF+/MAP+ giant cells expressed astrocyte-specific markers, e.g. GS and vimentin, had a bizarre shape, and one or several, eccentrically-located nuclei.

The majority of cytomegalic (67 of 73), as well as some normal-shaped neurons, in perituberal tissue were p-S6 immunoreactive (Figure 6h). However, we did not consider this staining property to be a reliable marker of a neuronal abnormality, since many neurons in neocortical and hippocampal gray matter in samples of brains obtained from temporal lobe epilepsy and other non-epilepsy surgeries revealed high levels of p-S6 immunoreactivity (unpublished data).

It is important to note that cytomegalic neurons were not a part of perituberal microtubers (found in 0/31 microtubers examined by double staining for NF/MAP2 and GFAP). In addition, we did not observe single giant cells near cytomegalic neurons (12 giant cells and 14 cytomegalic neurons examined in double immunostains for NF/MAP2 and p-S6). We also did not detect any significant alterations in NG2 cells or oligodendrocytephenotypes in microtubers (not shown), similar to our previous analysis of tubers [20].

### Astrocyte heterogeneity in cortical tubers

As emphasized above, one of the most prominent features of tubers was a high level of astrogliosis. In 7/16 tubers, severe astrogliosis completely occupied the tuber gray matter (to pia surface) and almost every astrocyte was devoid of GS and glutamate transporter (EAAT1 and EAAT2) immunoreactivity. In the other 9/16 tubers, we found that upper cortical layers (usually layer 1 and 2) were populated with protoplasmic astrocytes and many microtubers were located near the tubers (Figure 1a,b,c). Small “islands” of protoplasmic astrocytes were observed within tuber highly gliotic tissue that produced a patchy pattern due to intermingled gliotic and non-gliotic areas (Figure 1e, 7a). Because we did not make 3-D reconstructions of the whole tubers we cannot rule out the idea that these island-like areas of normal brain parenchyma were not in reality deep invaginations of perituberal, normal parenchyma.

**Figure 7:**
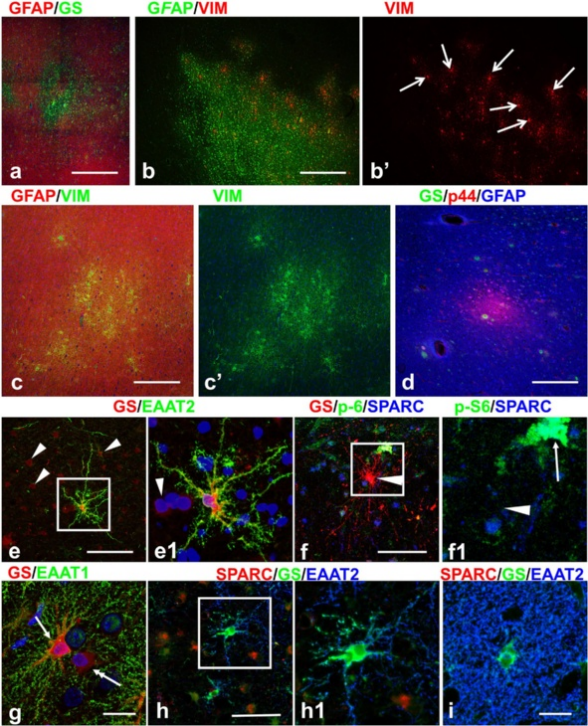
**Astrocyte heterogeneity in tubers. (a)** Variability in the levels of glutamine synthetase (GS, green) and GFAP (red) in the peripheral portion of a tuber. **(b)** Microtuber-like areas delineated with vimentin (VIM) immunostaining of astrocytes in a periphery of a tuber. VIM+ giants cells are indicated with arrows. **(c,d)** Astrocytes revealing features of reactive (high levels of VIM and expression of p44) are composed in small groups in the cenral parts of the tubers. **(e-h)** Unusual forms of astrocytes in ‘transitional’ areas at the tuber borders. **(e)** Fibrous-like astrocyte in gliotic tuber parenchyma expressing high levels of GS (red) and EAAT2 (green). Note: 1) main branches of the cell have only small spine-like processes; 2) neighboring gliotic astrocytes (arrowheads, shown only some) express minimal levels of GS and EAAT2. **(f)** Astrocyte with long straight processes and high level of GS does not show immunoreactivity for SPARC (arrowhead in **e1**). Giant cell is marked with arrow. **(g)** EAAT1 immunoreactivity is observed only in astrocyte (arrow) main branches. Note: neighboring astrocyte (double headed arrow) has minimal EAAT1 and GS. **(h)** Astrocyte with long main branches devoid of EAAT2 immunoreactivity in small leaf-like processes is SPARC immunonegative. **(i)** Normal protoplasmic astrocyte in normal gray matter. Note abundance of small leaf-like processes almost completely filling the neuropile between main cellular branches. Confocal microscopy double **(a,c,e,g)** and triple **(d,f,h,i)** immunostaining, counterstaining with Nissl in cases with double immunostaining. **b**’ and **c**’ represent split **b** and **c** images, respectively. **e1**, **f1**, and **h1** enlarged boxed area in **e**, **f**, and **g**, respectively. *scale bar*: 140 μm in **a-d**, 70 μm in **e**,**f**,**h**, 45 μm in **g**,**i**.

Although most tubers were composed of a homogeneous population of gliotic astrocytes, in some areas within tubers it was possible to discriminate groups of astrocytes displaying higher levels of vimentin, p-S6, and p44 than neighboring gliotic astrocytes (Figure 7b,c,d). These cells were grouped around giant cells and might correspond to microtubers with astrocytes that did not reach the “final stage of gliotic maturation” observed in neighboring highly gliotic tuber astrocytes. The average diameter of such groups was 329 ± 15.1 μm (n = 15; range: max 459.3 μm, min 236.7 μm), overlapping extensively with the dimensions of the perituberal microtubers. Indeed, these groups within tubers exceeded the average dimension of microtubers in the perituberal parenchyma by less than the size of one astrocyte domain (~100 μm, not including the area of overlapping processes from neighboring cells [21]).

**Figure 8:**
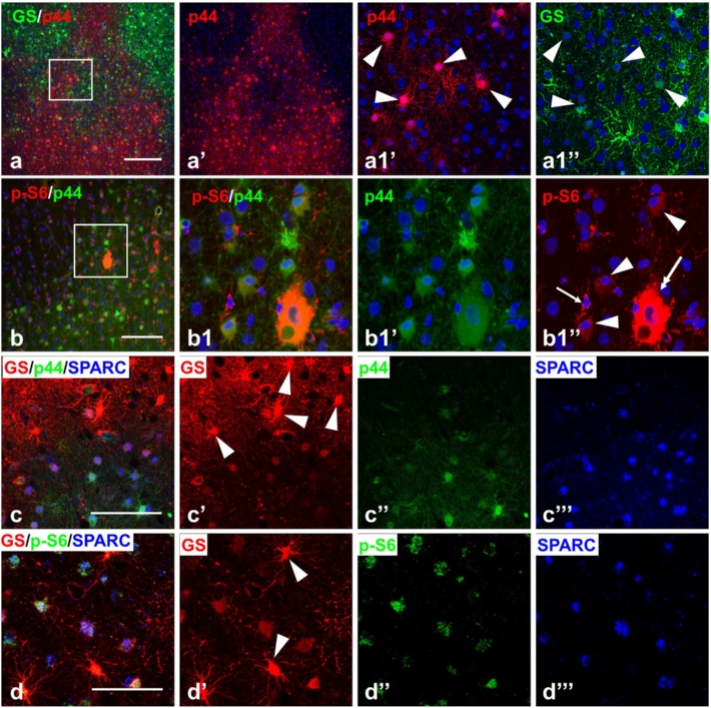
**Reactive features of astrocytes in the tubers. (a)** p44+ gliotic astrocytes delineate the border between gliotic and non-gliotic areas in the peripheral parts of the tuber and are segregated from GS+ protoplasmic astrocytes. Note that only astrocytes with low levels of GS expression are p44 immunopositive (arrowheads in **a1**’ and **a1**”). **(b)** p44 + astrocytes in transitional border area express p-S6 (arrowheads). Giant cell (double headed arrow) expresses p44 and p-S6. p-S6+ microglia (arrow) don’t show p44 immunoreactivity. **(c)** p44+ gliotic astrocytes express SPARC. Note that protoplasmic astrocytes are p44 and SPARC immunonegative (arrowheads). **(d)** SPARC+ astrocytes express p-S6. Note that the level of p-S6 expression in protoplasmic SPARC immunonegative astrocytes (arrowheads) is less than in SPARC+ astrocytes. Confocal microscopy, double immunostaining **(a,b)**, counterstaining with Nissl; triple immunostaining** (c,d)**. **a1**’, **a1**”, **b1**’, and **b1**”split images from enlarged inserts in **a** and **b**, respectively. **c**’, **c**”, **c**”’, **d**’, **d**”, and **d**”’ respectively split **c** and **d** images, respectively. Scale bar: 100 μm in **a**, 75 μm in **b**, **c**, **d**.

The phenotype of astrocytes located at the tuber border zones was different from that of astrocytes in the central part of the tubers, as well as from that of normal protoplasmic astrocytes in the neighboring gray matter. These ‘transitional’ areas, which we speculate represent developing gliosis in tubers, were populated with astrocytes that had characteristic features consistent with ongoing astrocyte changes. 1) Only in these areas were there unusual astrocytes with shapes of fibrous astrocytes (cells with long processes devoid of miniature leaf-like protrusions) and yet with high levels of GS, EAAT1, and EAAT2, which are characteristic features of protoplasmic astrocytes (Figure 7e-h). Taking into consideration that the majority of these unusual astrocytes did not show immunoreactivity for SPARC (Figure 7f,h), we considered them to be astrocytes with mixed protoplasmic and fibrous features. 2) Many astrocytes in these areas were immunopositive for phosphorylated p44 MAPK. These p44+ astrocytes showed minimal immunoreactivity for GS and glutamate transporters. (Figure 8a-c). 3) p-S6+ astrocytes predominated in these areas and often immunoreactivity for p-S6 colocalized with p44 and SPARC (Figure 8b,d). Both p44+ and p-S6+ astrocytes expressed SPARC and revealed low levels of GS and glutamate transporters, whereas astrocytes with high levels of GS and glutamate transporters were SPARC immunonegative (Figure 7f,h; 8c,d). Thus, many astrocytes in these “transition zones” display features of both protoplasmic and long-process astrocytes. These may well reflect a phenotype in transition.

The level of microglial activation (based on immunoreactivity for CD68 and Iba1) in these transitional areas was similar to that in tubers and significantly exceeded that of the non-gliotic areas surrounding tubers (not shown).

## Discussion

The consistent presence of tuber-specific abnormalities in epileptogenic MRI-normal perituberal cortex and subcortical white matter favors the thesis that brain pathology in TSC is much more widespread than is usually considered and includes other forms of pathological alterations besides the classical entities of cortical tubers, subependymal nodules, and giant cell astrocytomas [22-25]. Recent analysis of autopsied patients with TSC showed that small groups of giant cells and neurons, named ‘microtubers’, were found all over the neocortex and even in cerebellum [12,26]. Our definition of ‘microtuber’ is quite different because we include aberrant astrocytes as essential components of microtubers. Thus we have excluded isolated giant cells not surrounded with aberrant astrocytes from this definition. Widespread perituberal micropathology of clusters of giant cells and dysplastic neurons was also reported recently in a series of surgically resected TSC tissues [27]. These pathological alterations in the human brain in TSC resemble “diffuse” changes involving the whole brain observed in animal models of TSC [28-32] and thus may diminish critiques of these models based on the absence of prominent focal loci of aberrant brain architecture.

### Is perituberal tissue an epileptogenic area?

What is the functional significance of aberrant cellular elements in perituberal tissue? Do they participate in seizure initiation and/or progression? Seizure origin in TSC is the subject of much debate with several observations favoring perituberal tissue as a source of spontaneous epileptiform activity [5-9]. We found that perituberal tissue contains both microtubers and cytomegalic neurons, both of which may contribute to the epileptogenicity of this tissue. Electrophysiological analysis of tubers and cortical dysplasia has shown that only cytomegalic/dysplastic neurons revealed features of hyperexcitabilty, while giant/balloon cells were electrophysiologically silent [33]. Cytomegalic neurons in perituberal tissue may have similar or identical properties of their tuber counterparts and thus could potentially trigger ictal events. Similarly, the aberrant astrocytes that surround giant cells in “microtubers” are characterized by markedly diminished expression of EAAT1, EAAT2, and GS, proteins important for the uptake and metabolic conversion of glutamate to glutamine within astrocytes. We have found that similar astrocytes in tubers have deficient glutamate uptake [34]. Thus, even normal neurons located in microtubers may be predisposed to overexcitation because of the profound diminution of astrocytic glutamate clearance [35,36]. Our finding of microtubers and cytomegalic neurons in an epileptogenic hippocampal focus (Figure 6) also supports the thesis that these aberrant perituberal components might be the triggers of perituberal seizures in TSC.

### Microtubers and the derivation of tuber gliotic astrocytes

Despite great progress in the molecular biology of TSC, less is known about the origin and development of phenotypic features of abnormal cell types in the TSC brain. Analysis of microtubers may help to elucidate how tuber gliotic astrocytes develop.

Cortical tubers are composed of tightly packed gliotic astrocytes that make immunohistochemical analysis of individual cell shape and properties very complicated, if possible at all. In contrast, in microtubers where cells are not so densely packed, we identified two types of astrocytes: 1) astrocytes with long, straight, main processes lacking miniature leaf-like protrusions (the majority of astrocytes) and 2) protoplasmic astrocytes revealing features of reactive cells (a minority of cells). The first, based on morphology and immune profile, are similar to the CD44+ long-process astrocytes found at the pial surface and in white matter [17,21]. We propose that the majority of gliotic tuber astrocytes are aberrant CD44+ long process/fibrous-like astrocytes that due to abnormal development appear and replace the normal protoplasmic astrocytes in gray matter. Thus, due to such aberrant development, astrocytes that usually are located in white matter and the pial surface populate the gray matter.

Although long-process astrocytes in normal gray matter were described by Ramon y Cajal [37], their appearance and functions in gray matter remain mysterious. It should be remarked that while the divergent development of neurons and glial cells (including astrocytes) is well studied [38,39], there is little known about the divergent origins of protoplasmic, CD44+ long process, and fibrous astrocytes.

The appearance of reactive-like astrocytes in microtubers raises critical questions about the relationship between giant cells and neighboring astrocytes. Why are giant cells often but not always surrounded by reactive protoplasmic astrocytes? What may be the cause of these reactive-like changes in astrocytes? One possibility based on our data is that giant cells may release cytokines, e.g. TNFα that promote local reactive changes in astrocytes. As is well known, TNFα is a powerful trigger of reactive changes in astrocytes [19].

Many astrocytes in microtubers and in tubers showed a high level of immunoreactivity for activated (phosphorylated) p44 MAPK. It should be noted that overactivation of p44 was consistently observed and considered as an important, although unexplained, mechanism in TSC-related neoplasms [40]. p44 and mTOR pathways have close, although not completely understood, interrelations. p44 may inactivate the TSC1-TSC2 complex through phosphorylation of TSC2 [41]; while mTOR can provide negative (through p70 S6K1 and PI3K, [42]) or positive (through protein phosphatase 2A, [43]) feedback on p44. At this time, it remains unclear how p44 MAPK activation may be related to astrocyte changes in TSC.

The heterogeneity of the astrocyte population in microtubers and tubers shown in this study is in line with a recent detailed analysis of the glial and neuronal populations in tubers [15]. These authors and our previous work [20] defined several types of aberrant astrocytes in tubers, including reactive astrocytes. Reactive changes in astrocytes, e.g. expression of p-S6 and p44, can be used as a good indicator of acute ongoing cellular alterations [19,44].

### Are macrotubers built of microtubers?

Should microtubers be considered as elementary units or domains, which by consecutive aggregation create the main bodies of macrotubers? This “elementary unit” hypothesis is grounded largely on the anatomical observations: 1) rather homogeneous size of microtubers; 2) consistent association of microtubers with tuber borders; 3) appearance of microtuber-like areas with reactive astrocytes surrounding giant cells in tubers especially near their borders. We hypothesize that the CD44+ long-process astrocytes and the giant cells in individual microtubers comprise the progeny of one or more neural precursors in the embryonic ventricular zone (VZ) that have developed LOH at a TSC locus or have developed another mutation that interfere with their normal developmental program. VZ cells that remain heterozygous at the TSC 1 or 2 loci will generate normal cortical populations. Early genetic changes that give rise to large numbers of altered VZ cells may give rise to tubers, whereas late genetic changes, which give rise to one or only a few altered VZ cells may give rise to microtubers.

## Conclusion

In this study we have focused on astrocyte changes at the perituberal border and in microtubers. This focus has allowed us to define tuber borders. It has also allowed us to define microtubers as units composed not only of giant cells but also of aberrant gliotic astrocytes which constitute and define features of macrotubers, e.g. hardness of the tubers. Such an approach provides novel insights into the cellular abnormalities in MRI normal epileptogenic perituberal tissue in TSC, and opens new perspectives to consider in the therapeutic and surgical treatment of TSC related epilepsy.
